# Profiling the immune landscape in mucinous ovarian carcinoma

**DOI:** 10.1016/j.ygyno.2022.10.022

**Published:** 2022-11-08

**Authors:** Nicola S. Meagher, Phineas Hamilton, Katy Milne, Shelby Thornton, Bronwyn Harris, Ashley Weir, Jennifer Alsop, Christiani Bisinoto, James D. Brenton, Angela Brooks-Wilson, Derek S. Chiu, Kara L. Cushing-Haugen, Sian Fereday, Dale W. Garsed, Simon A. Gayther, Aleksandra Gentry-Maharaj, Blake Gilks, Mercedes Jimenez-Linan, Catherine J. Kennedy, Nhu D. Le, Anna M. Piskorz, Marjorie J. Riggan, Mitul Shah, Naveena Singh, Aline Talhouk, Martin Widschwendter, David D.L. Bowtell, Francisco J. Candido dos Reis, Linda S. Cook, Renée T. Fortner, María J. García, Holly R. Harris, David G. Huntsman, Anthony N. Karnezis, Martin Köbel, Usha Menon, Paul D.P. Pharoah, Jennifer A. Doherty, Michael S. Anglesio, Malcolm C. Pike, Celeste Leigh Pearce, Michael L. Friedlander, Anna DeFazio, Brad H. Nelson, Susan J. Ramus

**Affiliations:** a School of Clinical Medicine, UNSW Medicine and Health, University of NSW Sydney, Sydney, New South Wales, Australia; b Adult Cancer Program, Lowy Cancer Research Centre, University of NSW Sydney, Sydney, New South Wales, Australia; c The Daffodil Centre, The University of Sydney, A Joint Venture with Cancer Council New South Wales, Australia; d Trev & Joyce Deeley Research Centre, British Columbia Cancer Agency, Victoria, BC, Canada; e The Walter and Eliza Hall Institute of Medical Research, Parkville, Victoria, Australia; f Centre for Cancer Genetic Epidemiology, Department of Oncology, University of Cambridge, Cambridge, UK; g Department of Gynecology and Obstetrics, Ribeirão Preto Medical School, University of São Paulo, Ribeirão Preto, Brazil; h Cancer Research UK Cambridge Institute, University of Cambridge, Cambridge, UK; i Canada’s Michael Smith Genome Sciences Centre, BC Cancer, Vancouver, BC, Canada; j British Columbia’s Gynecological Cancer Research Team (OVCARE), University of British Columbia, BC Cancer, and Vancouver General Hospital, Vancouver, BC, Canada; k Program in Epidemiology, Division of Public Health Sciences, Fred Hutchinson Cancer Center, Seattle, WA, USA; l Peter MacCallum Cancer Centre, Melbourne, Victoria, Australia; m Sir Peter MacCallum Department of Oncology, The University of Melbourne, Parkville, Victoria, Australia; n Center for Bioinformatics and Functional Genomics and the Cedars Sinai Genomics Core, Cedars-Sinai Medical Center, Los Angeles, CA, USA; o MRC Clinical Trials Unit, Institute of Clinical Trials & Methodology, University College London, London, UK; p Department of Pathology and Laboratory Medicine, University of British Columbia, Vancouver, BC, Canada; q Department of Histopathology, Addenbrooke’s Hospital, Cambridge, UK; r Centre for Cancer Research, The Westmead Institute for Medical Research, Sydney, New South Wales, Australia; s Department of Gynaecological Oncology, Westmead Hospital, Sydney, New South Wales, Australia; t The University of Sydney, Sydney, New South Wales, Australia; u Cancer Control Research, BC Cancer, Vancouver, BC, Canada; v Department of Obstetrics and Gynecology, Division of Gynecologic Oncology, Duke University Medical Center, Durham, NC, USA; w Department of Pathology, Barts Health National Health Service Trust, London, UK; x Department of Anatomical Pathology, Vancouver General Hospital, Vancouver, Canada; y Department of Obstetrics and Gynecology, University of British Columbia, Vancouver, BC, Canada; z EUTOPS Institute, University of Innsbruck, Innsbruck, Austria; aa Epidemiology, School of Public Health, University of Colorado, Aurora, CO, USA; ab Community Health Sciences, University of Calgary, Calgary, AB, Canada; ac Division of Cancer Epidemiology, German Cancer Research Center (DKFZ), Heidelberg, Germany; ad Computational Oncology Group, Structural Biology Programme, Spanish National Cancer Research Centre (CNIO), Madrid, Spain; ae Department of Epidemiology, University of Washington, Seattle, WA, USA; af Department of Molecular Oncology, BC Cancer Research Centre, Vancouver, BC, Canada; ag Department of Pathology and Laboratory Medicine, UC Davis Medical Center, Sacramento, CA, USA.; ah Department of Pathology and Laboratory Medicine, University of Calgary, Foothills Medical Center, Calgary, AB, Canada; ai Huntsman Cancer Institute, Department of Population Health Sciences, University of Utah, Salt Lake City, UT, USA; aj Department of Epidemiology and Biostatistics, Memorial Sloan-Kettering Cancer Center, New York, NY, USA; ak Department of Population Health and Public Health Sciences, Keck School of Medicine, University of Southern California Norris Comprehensive Cancer Center, Los Angeles, CA, USA; al Department of Epidemiology, University of Michigan School of Public Health, Ann Arbor, MI, USA; am Nelune Comprehensive Cancer Centre, Prince of Wales Hospital, Sydney, New South Wales, Australia; an Gynaecological Cancer Centre, Royal Hospital for Women, Sydney, New South Wales, Australia

**Keywords:** Mucinous ovarian carcinoma, Immune infiltrate, Rare histotype

## Abstract

**Objective.:**

Mucinous ovarian carcinoma (MOC) is a rare histotype of ovarian cancer, with low response rates to standard chemotherapy, and very poor survival for patients diagnosed at advanced stage. There is a limited understanding of the MOC immune landscape, and consequently whether immune checkpoint inhibitors could be considered for a subset of patients.

**Methods.:**

We performed multicolor immunohistochemistry (IHC) and immunofluorescence (IF) on tissue microarrays in a cohort of 126 MOC patients. Cell densities were calculated in the epithelial and stromal components for tumor-associated macrophages (CD68+/PD-L1+, CD68+/PD-L1−), T cells (CD3+/CD8−, CD3+/CD8+), putative T-regulatory cells (Tregs, FOXP3+), B cells (CD20+/CD79A+), plasma cells (CD20−/CD79a+), and PD-L1+ and PD-1+ cells, and compared these values with clinical factors. Univariate and multivariable Cox Proportional Hazards assessed overall survival. Unsupervised k-means clustering identified patient subsets with common patterns of immune cell infiltration.

**Results.:**

Mean densities of PD1+ cells, PD-L1− macrophages, CD4+ and CD8+ T cells, and FOXP3+ Tregs were higher in the stroma compared to the epithelium. Tumors from advanced (Stage III/IV) MOC had greater epithelial infiltration of PD-L1− macrophages, and fewer PD-L1+ macrophages compared with Stage I/II cancers (*p* = 0.004 and *p* = 0.014 respectively). Patients with high epithelial density of FOXP3+ cells, CD8+/FOXP3+ cells, or PD-L1− macrophages, had poorer survival, and high epithelial CD79a + plasma cells conferred better survival, all upon univariate analysis only. Clustering showed that most MOC (86%) had an immune depleted (cold) phenotype, with only a small proportion (11/76,14%) considered immune inflamed (hot) based on T cell and PD-L1 infiltrates.

**Conclusion.:**

In summary, MOCs are mostly immunogenically ‘cold’, suggesting they may have limited response to current immunotherapies.

## 1. Background

Mucinous ovarian cancer (MOC) is a rare histotype of epithelial ovarian cancer with poor outcomes at advanced stage and a relatively poor response to platinum-based chemotherapy [1]. A prior study demonstrated that patients (MOC *n* = 343) with moderate levels of CD8+ tumor-infiltrating lymphocytes (TILs) (3–19 per high powered field) had better overall survival in MOC compared with no CD8+ TILs [Hazard ratio (HR) 0.56 (95% confidence interval (CI) 0.34–0.93), *p* = 0.04], with stronger associations observed in high-grade serous and endometrioid ovarian cancers [2]. However, beyond this single study, we have only a rudimentary understanding of the antitumor immune response in MOC, and consequently whether there may be a role for immune checkpoint inhibitors in a subset of patients.

In the context of immune checkpoint inhibition, current biomarkers thought to predict response include high tumor mutational burden, mismatch repair deficiency (MMRd)/microsatellite instability, high CD8+ T cell density, and PD-L1 expression [3]. Ovarian cancers in general have a lower tumor mutational burden relative to immunotherapy-responsive cancers such as melanoma or non-small cell lung cancer [4–6]. High-grade serous tubo-ovarian carcinoma (HGSC) typically has high copy number alterations rather than point mutations [7] and it is unclear whether this explains the low response to immune therapies seen to date (10–15% response rate) [8]. In HGSC, the C2-IMM molecular subtype is characterized as immunoreactive, and patients with these tumors have better survival outcomes [9,10]. Clear cell ovarian carcinoma may be more amenable to immune therapy which could potentially relate to *ARID1A* loss [3]. In a series of 184 MOCs, <1% of tumors had high tumor mutational burden or MMRd, which bodes poorly for single agent immune checkpoint inhibition [11].

Phenotypically, MOC may share an ‘intestinal’ histological appearance with gastrointestinal cancers. In terms of mutation profile [12], and gene expression profiles [13], MOC are very similar to upper gastrointestinal cancers, and microsatellite (MSI) high colorectal cancers with high CD8 TILs (associated with high TMB) were reported to have a 70% response to PD-L1 blockade [14]. In the clinical trial KEYNOTE-158 assessing efficacy of pembrolizumab (anti- PD-L1) in non-colorectal MSI high/MMRd cancers (*n* = 233), objective response rates were 45.8% in gastric, 42.1% in small intestine and 40.9% in cholangiocarcinoma, and 18.2% in pancreatic cancers [15]. By contrast, unselected pancreatic cancers have poor response to PD-1/PD-L1 blockade [16].

Given the paucity of data relating to the immune landscape in MOC, we used multi-color immunohistochemistry (IHC) and immunofluorescence (IF) to characterise the immune infiltrate in a 126-case cohort of patients with MOC, and examined associations between immune cell densities, clinical features, and survival. As therapeutic approaches targeting different features of the immune system develop, we aimed to shed light on the immune landscape in MOC to provide guidance for future studies considering immune-based therapies for these patients.

## 2. Methods

### 2.1. Patient cohort

Patient samples came from eight sites (cases-control studies and institutional biobanks) that contributed to the international Ovarian Tumor Tissue Analysis (OTTA) consortium [17] (Supplementary Table S1) and tissue microarrays (TMA) that were submitted for the Multidisciplinary Ovarian Cancer Outcomes Group study [18]. Samples were eligible for inclusion with a confirmed diagnosis of primary mucinous ovarian carcinoma. Cases underwent single slide review to confirm diagnosis and to classify the pattern of invasion (infiltrative/expansile, *n* = 95). The majority (*n* = 112) also had data available from central pathology review from IHC-based histotyping in prior OTTA studies [19–21]. Metastases, ‘seromucinous’ tumors (now considered endometrioid) [22] and mucinous borderline ovarian tumors were excluded. The total number of eligible MOCs across the TMAs was 151 cases before QC exclusions. Cores on TMAs were 0.6–1.0 mm, taken from a representative area selected by the study site pathologist. For consistency across studies a standard 20× image was analyzed per core which was approximately 0.6 mm. We stained and scanned TMAs by multi-color brightfield IHC using two panels (CD3/CD8 for T cells and CD20/CD79a for B cells), and by multi-color IF (CD68/PD-L1/PD-1 for macrophages and the PD-1/PD-L1 axis and, FOXP3/CD8 for Tregs). All panels also included pan-Cytokeratin to distinguish tumor epithelium from stroma (Supplementary Table S2). Antibodies used for multiplex Brightfield IHC, and multi-color immunofluorescence are detailed in Supplementary Table S3.

### 2.2. Brightfield IHC – tissue and cell segmentation

Manual inspection of images was used to quantify areas of tumor epithelium, stroma, and other (e.g. mucin), rounded to the nearest 5%. Cores were excluded if the proportions of both epithelium and stroma were <25% each, if there was folding in the core, or due to the presence of non-specific staining. Cell infiltrates were manually quantified as raw counts. The area in mm^2^ was calculated as 0.365 x region area, and the cell density computed as count/area. For samples with multiple cores, an average was taken to give one density value per cell type per case. Supplementary Fig. 1 shows an example of images from both methods.

### 2.3. Multi-color immunofluorescence (IF)

QuPath (REF v. 0.2_m2) was used to quantify immune cells in multi-color IF images. Briefly, semi-automated tissue segmentation was performed to the nearest μm^2^ as tumor epithelium or stroma based on intensity of pan-Cytokeratin expression, and annotations were manually reviewed and corrected to exclude mucinous regions from stromal annotations. Cells were detected using QuPath’s watershed detection algorithm and random forest classifiers trained for each panel to classify cell phenotypes of interest. Cores were excluded as per the brightfield criteria above, and cell densities for analysis calculated as the number of cells observed in each mm^2^.

### 2.4. Data preparation and analyses

Due to variability in staining intensity between studies (Supplementary Figs. 2–4), batch correction by study site was performed using ComBat-seq [23]. This resulted in the exclusion of two studies with too few cases (1 and 2 respectively) for batch correction. Data were log10 (x + 1) transformed for analysis. Differences in mean cell densities between groups were assessed using Welch’s *t*-test. The association between cell densities and overall survival (OS) was estimated using Cox proportional hazards, with right censoring at 10 years, and and left truncation of prevalent cases to guard against survival bias of cases entering the study at any length of time after diagnosis. Survival time was measured from study entry until death or end of follow-up. Multivariable analysis adjusted for age and tumor stage and stratified by study site. The proportional hazards assumption was tested using the cox.zph function in the survival package in R. Unsupervised k-means clustering was used to group cases with similar immune cell densities, and the Kaplan-Meier survival method was applied to visualise survival curves for the resulting clusters. Expression data for the mismatch repair (MMR) proteins (MLH1, MSH2, MSH6, PMS2) by IHC was available for a small subset of cases (*n* = 23), to explore associations between MMR status and immune cell infiltrates. Statistical significance was considered where *p* < 0.05 and all analyses were performed using R v4.2.0.

## 3. Results

### 3.1. Patient and tumor characteristics and immune infiltrate composition

There were 126 patients (188 cores) with evaluable data after 25 were excluded due to core drop-out or inadequate staining or imaging. For some patients, not all cell phenotypes were available due to excluded cores (Supplementary Table S4). A total of 76 patients had complete immune phenotypic data involving all evaluated markers. Patient characteristics are shown in Table 1. The median age at diagnosis was 54. Most patients were early Stage (I or II) at diagnosis (82%, 97/118), 16% (15/95) had an infiltrative pattern of invasion, and only 4% (5/112) of patients with known grade had grade 3 MOC. The composition of tumor epithelium and stroma across cores was highly variable (Table 1).

**Fig. 1.**
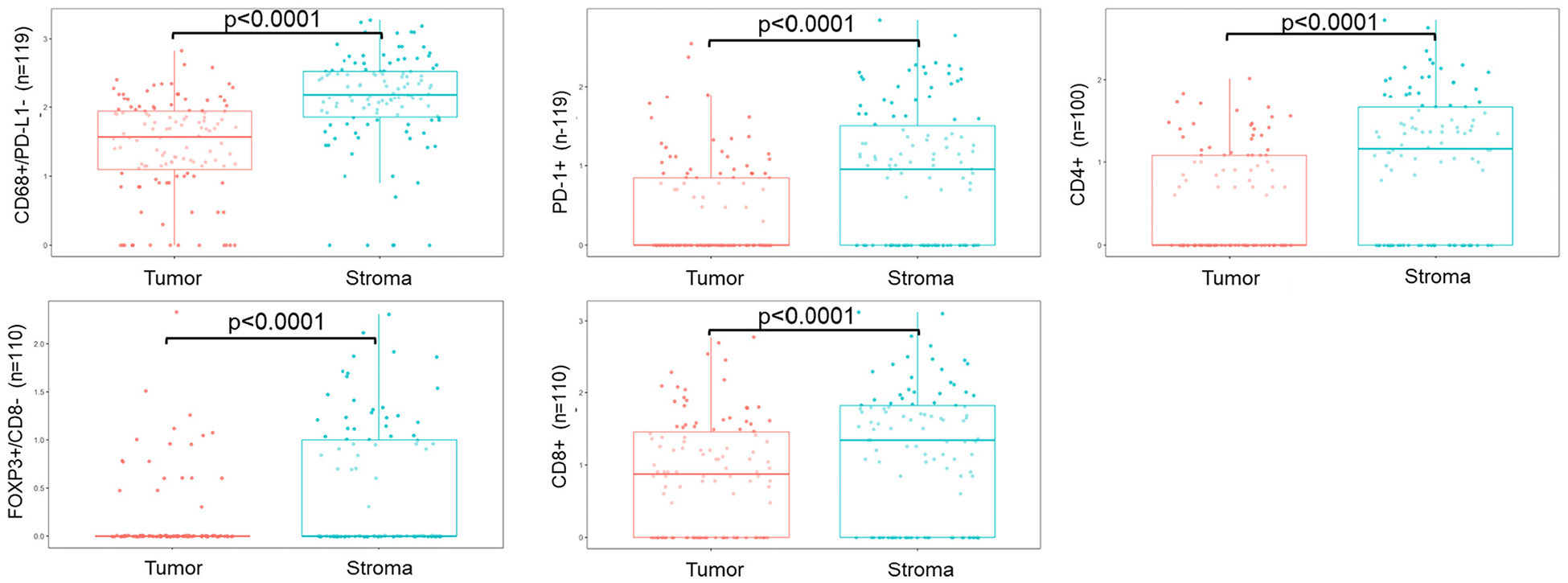
Boxplots of statistically significantly different densities in immune phenotypes by tumor epithelium/stromal regions. Difference in mean density calculated using Welch’s *t*-test.

The overall mean tumor epithelial region was 43% (standard deviation 16%, range 7–76%) and mean stromal region was 22% (standard deviation 15%, range 1–67%), the remaining area was composed of mucin, no cells, or lymphovascular space. The average epithelial:stromal composition of cores ranged from 1.4 to 2.1 between panels due to natural heterogeneity in tumor samples and the use of two different techniques for tissue segmentation (manual for brightfield versus semi-automated for immunofluorescence). However, there was no difference in the proportion of patients with different stage and grade between panels included in each analysis (Supplementary Table S5). Mean densities of PD1+ cells, PD-L1−/CD68+ macrophages, CD8+ T cells, FOXP3+ Tregs, and presumptive CD4+ T cells (CD3+/CD8−; referred to hereafter as CD4+ T cells) were all higher in stroma compared to epithelium (Fig. 1). There was no difference in the mean density of PD-L1+/CD68+ macrophages, PD-L1+ cells, CD8+/FOXP3+ cells, CD79a + B cells and plasma cells or CD20+ B cells between epithelial and stromal areas (Supplementary Fig. S5). All samples with complete MMR IHC data (*n* = 23) had preserved staining, therefore were MMR proficient.

### 3.2. PD-L1 expression by tumor-associated macrophage populations differs in low and high stage MOC

**Fig. 2.**
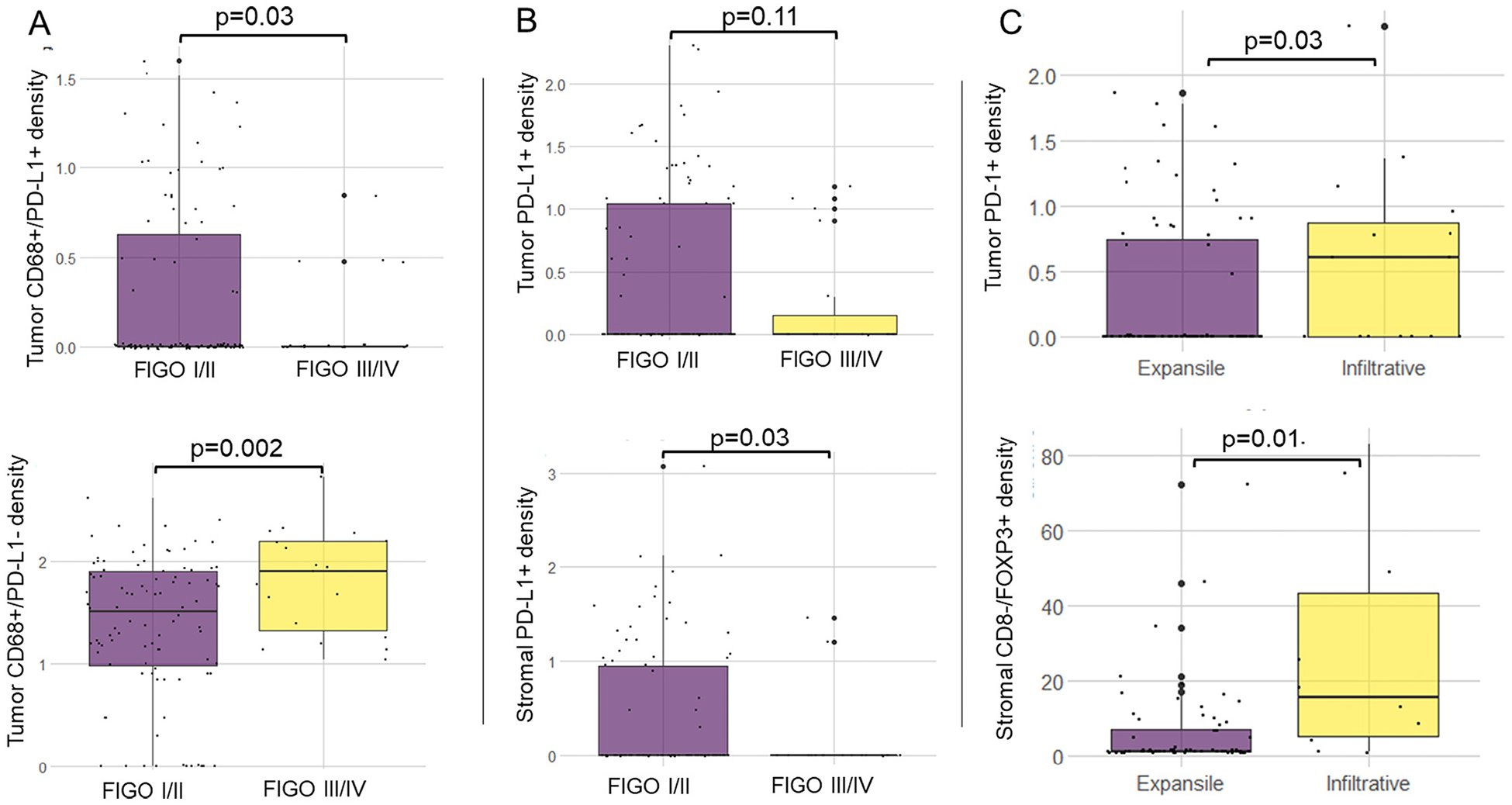
Differences in immune cell density by stage (*n* = 115) and pattern of invasion (*n* = 90). Panel A: tumor epithelial CD68+/PD-L1+ TAMs (top), tumor epithelial CD68+/PD-L1− TAMs (bottom). Panel B: tumor epithelial PD-L1+ cells (top); stromal PD-L1+ cells (bottom). Panel C: tumor epithelial PD-1+ cells (top); stromal CD8-/FOXP3+ cells (bottom). Differences in mean density calculated using Welch’s *t*-test.

Tumors from advanced stage disease (FIGO Stage III/IV) had a higher density of PD-L1− macrophages (CD68+/PD-L1−; *p* = 0.004) and lower density of PD-L1+ macrophages (CD68+/PD-L1+; *p* = 0.014) compared to those from early stage disease (Fig. 2, Table 2). Early stage tumors had higher mean density of PD-L1+ cells in the stroma compared with advanced stage MOC (*p* = 0.003). Tumors with an infiltrative pattern of invasion had a higher density of PD-1+ cells in the tumor epithelium (*p* = 0.03), and higher CD8−/FOXP3+ cells in the stroma (p = 0.01, Fig. 2, Table 2). This finding was not replicated when we restricted analysis to FIGO Stage I cases only (*n* = 61, Supplementary Table S6). The only cell type to differ significantly by grade was the stromal density of CD4 T cells (CD3+/CD8+), which were higher in Grade 1 tumors, compared with Grade 2 and 3 (*p* < 0.01, Table 2).

### 3.3. Associations between immune cell densities and survival

On univariate analysis, high epithelial densities of CD8−/FOXP3+ cells and CD8+/FOXP3+ cells were associated with poorer overall survival (OS), (hazard ratio (HR) 2.20 [95% confidence interval (CI) 1.07–4.51], *p* = 0.032), and (HR 7.90, [95% CI 2.52–24.79], *p* < 0.001) respectively, Table 3. High epithelial PD-L1 negative macrophages (CD68+/PD-L1−), were also associated with poorer OS on univariate analysis (HR 1.78 [95% CI 1.03–3.08], *p* = 0.039). In contrast, increased density of epithelial CD79a + plasma cells was associated with improved OS, (HR 0.53 [0.30–0.94], *p* = 0.03.) There were no associations observed for the stromal infiltrates, and no association between CD8+ TIL density and OS was observed (HR 1.01 [95% CI 0.64–1.59], *p* = 0.957). None of the associations remained significant on multivariable analysis, with adjustment for age and stage and stratification by study site.

### 3.4. Mucinous ovarian carcinomas are largely immunologically ‘cold’

**Fig. 3.**
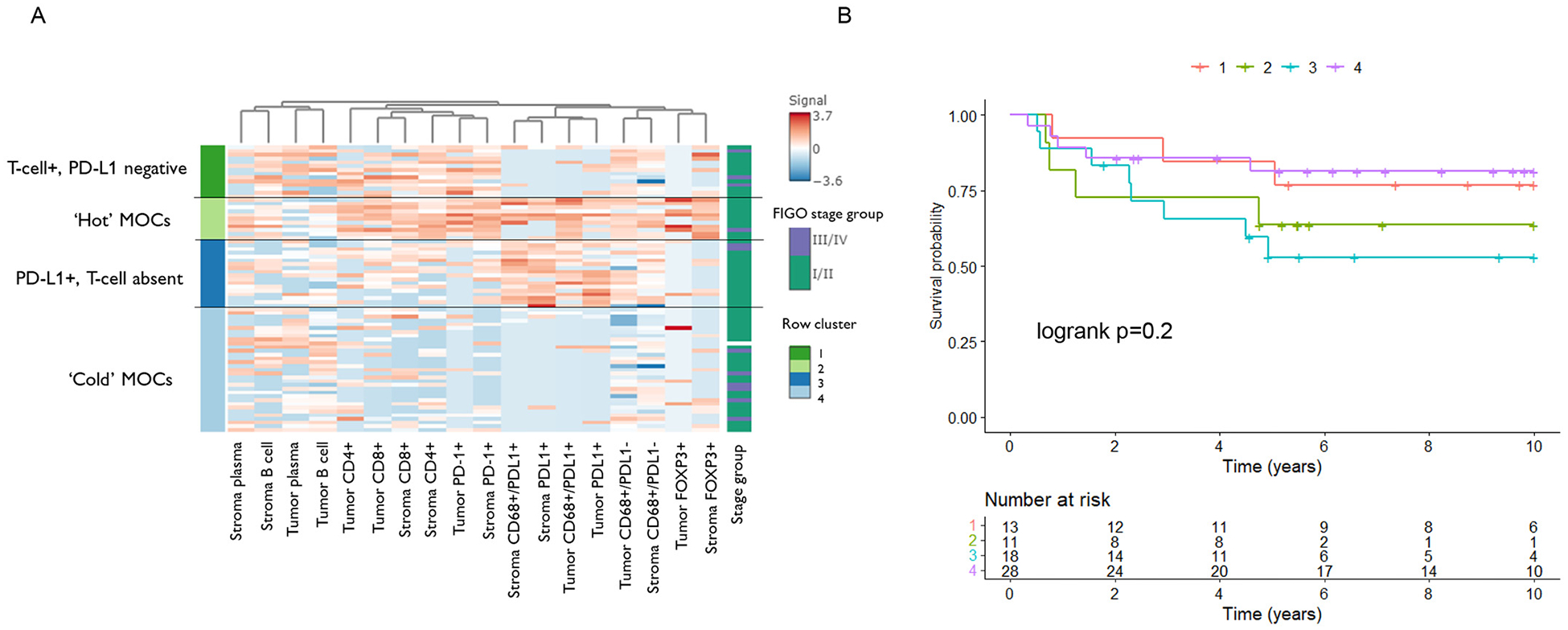
A) Unsupervised kmeans clustering of cell densities in tumor epithelium and stroma (*n* = 76); B) Kaplan-Meier survival curves of overall survival by cluster groups 1‒4 (*n* = 70). Differences between survival curves calculated using the logrank test.

Unsupervised clustering segregated samples into 4 clusters based on immune cell densities in the epithelium and stroma (Fig. 3A). Cluster 1 samples (*n* = 14) were characterized by epithelial and stromal T cell presence and absence of PD-L1, whereas cluster 3 (*n* = 18) showed the reverse, with T cell absence but presence of PD-L1+ cells. Cluster 2 (n = 11) was considered immune ‘hot’ (T cell+ and PD-L1+). By contrast, cluster 4 was the largest (*n* = 33) and represented an immunogenically ‘cold’ group, characterized by absence of T-cell infiltrate and PD-L1 negativity. There was no difference in the overall survival outcomes across clusters, logrank *p* = 0.2 [Fig. 3B, Supplementary Table S7). We performed a sensitivity analysis restricting the clustering to only cases of FIGO stage IC or above, to represent the patient population most likely to have been indicated for adjuvant treatment (Supplementary Fig. S6). The immune cell phenotypes of the 4 groups were replicated and 6/33 (18%) clustered in the immune ‘hot’ group. The MMR status was known for four of the patients in this hot cluster and all were proficient.

## 4. Discussion

Given the paucity of treatment options for patients with MOC, it is necessary to get an understanding of whether there may be a subgroup(s) of patients who might benefit from immunotherapy. We observed significant differences in the tumor-associated macrophage (TAM) populations between low and high stage disease suggestive of better immune control in tumors diagnosed at an early stage. Tumors from patients with advanced stage disease had significantly fewer PD-L1+ macrophages and more PD-L1− macrophages. Given that macrophages can be broadly categorized into two functionally different subtypes, one possibility is that PD-L1+ macrophages have a more antitumorigenic M1 phenotype, including secretion of pro-inflammatory cytokines, where PD-L1− TAMs could represent M2, suppressing the immune response and facilitating tumor progression. Indeed, in high-grade serous tubo-ovarian cancer, PD-L1+ TAMs have been shown to be independently associated with improved OS [24] and the M2 macrophage phenotype is associated with poor survival [25]. Studies analyzing the association between TAM infiltrates and prognosis have used various measures (i.e. CD163, CD68, M1/M2 ratio) and a meta-analysis in ovarian cancer (histologies combined) found that only the M1/M2 ratio was significant for both overall and progression free survival [26]. A high M1/M2 ratio had better outcomes, whereas there was no association between CD68+ TAMs and OS (HR = 0.99, 95% CI 0.88–1.11, *p* = 0.859). [26] TAM targeted therapies are a newer area under investigation [27], and in the context of these findings for MOC, PD-L1 negative TAMs may be an appropriate target for depletion or reprogramming to a more anti-tumorigenic M1 phenotype. Similarly tumors with an infiltrative pattern of invasion had higher tumor epithelial PD-1+ cells, and higher stromal FOXP3+ Tregs. An infiltrative pattern of invasion is associated with a poorer prognosis [13], potentially involving an environment of T cell exhaustion/suppression.

We showed on univariate analysis only that high epithelial density of FOXP3+ cells was associated with poorer overall survival. A meta-analysis examining the association of FOXP3+ cells with survival across different tumor types has shown varying directions of effect [28], and additional findings of a favorable association have been shown by IHC in ER-negative breast cancer [29] and high-grade serous tubo-ovarian cancer [30]. A large mRNA expression profiling study in high-grade serous ovarian cancer found that patients with high tumor expression of *FOXP3* had better survival (HR 0.93, 95% CI 0.89–0.97, *p* = 1.36E-03) [31]. These contrasting results make it difficult to draw conclusions in MOCs, however the biology is consistent with Tregs having an inhibitory effect on the immune response, supressing T effector cells, and promoting tumor growth. Having higher levels of FOXP3+ Tregs in MOC could therefore have an immunosuppressive effect. Current therapeutic suggestions include inhibiting FOXP3+ cells to allow immune therapies to work [32,33].

On univariate analysis, we observed better survival in patients with higher epithelial CD79a plasma cells but not stromal; and no significant association was seen for CD20 B cells. A prior study in HGSC showed that plasma cells were more common in the stromal rather than epithelial regions, correlated with other TIL-T and TIL-B subsets, and appeared to enhance their survival association [34]. B cells and plasma cells are associated with a favorable prognosis across multiple cancer types [35]. Studies have indicated a co-operative environment between plasma cells and other TIL subsets that warrants further investigation. We did not observe an association between high CD8 TILs and prognosis as previously described in the larger cohort of 343 cases that was scored with a 4-point ordinal scoring system [2], but we showed lower densities of CD8+ T cells in the tumor epithelium compared to stroma implying little CD8+ T cell/tumor antigen interaction in MOC. Consistent with low levels of mismatch repair deficiency previously reported [11,36], none of the small subset of cases in our cohort (*n* = 23) had loss of MMR.

Our clustering results demonstrated that only 14% of MOCs (11/76) could be considered immunologically ‘hot’ based on T cell and PD-L1+ cell infiltrates (cluster 3, Fig. 3). Given the response to immune checkpoint inhibitors such as anti-PD-1 therapies is thought to be aided by the presence of existing T cells, our clustering suggests that at best this could benefit up to 33% of patients (25/76, clusters 1 and 2). Despite this, only 5% (4/76) had T cell positivity *and* were advanced stage – arguably the subset in need of therapeutic options. These results and the distribution by stage suggests that most high stage MOCs appear immunologically cold and there were no significant differences in OS between clusters. From a treatment perspective, it is difficult to conclude from these data that there is a cohort of patients with advanced stage MOC for whom PD-1/PD-L1 blockade would be effective. That said, this suggestion should be interpreted with caution, as our cohort did not contain samples from patients treated with checkpoint blockade or any other form of immunotherapy.

Many similarities have been drawn between GI cancers and MOC such as the intestinal histological phenotype [37], mutation profiles, (e.g. high rates of *KRAS* mutations) [12], gene expression profiles [13], and the fact that GI metastases can often be a diagnostic challenge for pathologists [38]. Despite this, there are limited data comparing the immune infiltrate between these tumor types. In the context of the immune landscape, data from pancreatic cancer suggests that high tumor infiltration of CD4+ and CD8+ T cells, low Tregs, and a high M1/M2 macrophage ratio are all associated with improved survival in pancreatic cancer [39]. Gastric cancers have shown adverse outcomes with high tumor FOXP3+ Tregs [40], similar to the finding in this study. With respect to PD-1/PD-L1, we have previously shown that tumor mRNA expression of PD-1 was lower in MOC compared to upper GI (pancreatic and gastric) and lower GI (colorectal and appendiceal) tumors. PD-L1 was lower in MOC compared to upper GI and no different to lower GI [13]. Some comparisons within GI cancers at the histological level have shown that mucinous compared with non-mucinous gastric cancers had higher PD-L1 expression [41], and mucinous differentiation in colorectal cancer appeared to be associated with lower tumor infiltrating lymphocytes compared with more common adenocarcinoma [42]. While there are suggestions that the best therapeutic approach for MOC points to basket clinical trials with upper GI cancers based on shared molecular features [43], whether there are enough similarities immunologically for an immune-related treatment arm remains unclear.

The strength of this study is its relative size given the rarity of MOC, and the broad assessment of different types of lymphocytes as well as macrophages in the one analysis. One limitation is the use of tissue microarrays (TMA) rather than whole sections. It is already known that MOCs are large, heterogeneous tumors, often containing areas of borderline or benign neoplasia. The assessment of stromal areas could be biased, given that the cores are selectively punched to represent areas with high tumor epithelium area. The samples were collected over different time periods and TMAs were constructed in different centres, therefore despite performing a batch correction before analysis, there is the possibility of uncontrolled batch effects. For example, we observed high PD-L1 densities in one particular study which could have an effect on the results; however, sensitivity analysis removing this study found similar results (Supplementary Table S8). We do not believe that there were systematic issues in the exclusion of samples from clustering analysis due to incomplete data across panels. One additional limitation was the lack of adjuvant treatment data, as well as the high proportion of early stage MOC cases who would not have an indication for further treatment. The sensitivity analysis restricting clustering to stage IC or higher (patients more likely to have adjuvant treatment) was performed to account for this.

Overall we observed higher levels of PD-L1− TAMs (M2) in high stage compared to low stage MOC, higher density of T-regulatory FOXP3+ cells in the stroma and an association of epithelial FOXP3+ cells with shorter survival, suggesting a more immune suppressive microenvironment in high stage/high risk MOC. Based on our current understanding of features that make cancers amenable to immunotherapy, MOCs lack mismatch repair deficiency, and are largely ‘cold’ with respect to immune cell infiltrates. The implications of this in a treatment context are untested; one of the few trials for which MOC is eligible – DART, a phase II trial of the immune checkpoint inhibitors Nivolumab and Ipilimumab in patients with rare tumors – has not published any response data in MOC to date. Based on our findings, if immune-based therapy were to be pursued in MOC, tailored approaches for different subgroups of MOC patients may be necessary. Additional options could explore more novel alternatives such as T cell receptor cell-based immunotherapy, theoretically considered a better option for ‘cold’ tumor types [5], even in a low TIL environment as observed here in MOC.

## Supplementary Material

1

2

## Figures and Tables

**Table 1:** Clinical characteristics of the cohort.

Total evaluable n	126
Age (median)	54
Stage I/II^a^	97 (82)
Stage III/IV^a^, n (% of known)	21 (18)
Stage unknown	8
Expansile pattern of invasion^a^	80 (84)
Infiltrative pattern of invasion^a^	15 (16)
Pattern of invasion unknown	31
Grade 1^a^	53 (47)
Grade 2^a^	54 (48)
Grade 3^a^	5 (4)
Grade unknown	14
Tumor area % (mean, standard deviation)^b^	43,16
Tumor area % (range)	7–76
Stromal area % (mean, standard deviation)^b^	22,15
Stromal area % (range)	1–67

a n (% of known).

b Calculated across multiple cores.

**Table 2:** Associations between immune cell phenotypes and clinical variables.

	Stage I/II vs. III/IV, p-value	Pattern of invasion	Grade (1 vs. 2/3), p-value
		(expansile vs. infiltrative), p-value	
CD68+ PDL1−^a^			
Epithelium	**<0.01**	0.53	0.21
Stroma	0.08	0.38	0.26
CD68+ PDL1+^a^			
Epithelium	**0.03**	0.31	0.87
Stroma	0.45	0.26	0.87
PDL1+^a^			
Epithelium	0.11	0.86	0.83
Stroma	**0.03**	0.26	0.98
PD1+^a^			
Epithelium	0.77	**0.03**	0.12
Stroma	0.08	0.20	0.78
CD3+/CD8+ (CD4+) T cells^b^			
Epithelium	0.29	0.52	0.69
Stroma	0.17	0.38	**<0.01**
CD8+ T cells^a^			
Epithelium	0.37	0.99	0.83
Stroma	0.60	0.22	0.12
CD8+ FOXP3+^a^			
Epithelium	0.24	0.47	0.71
Stroma	0.52	0.76	0.64
CD8− FOXP3+^a^			
Epithelium	0.58	0.06	0.99
Stroma	0.50	**0.01**	0.41
CD20+ B cell^b^			
Epithelium	0.58	0.84	0.23
Stroma	0.35	0.60	0.85
CD79a + plasma cell^b^			
Epithelium	0.51	0.36	0.90
Stroma	0.36	0.55	0.47

*p*-values, calculated using Welch’s t-test.

a Immunofluorescence Stage I/II *n* = 97, Stage III/IV *n* = 21, expansile *n* = 77, infiltrative *n* = 15, Grade 1 *n* = 52, Grade 2/3 *n* = 58.

b Immunohistochemistry Stage I/II *n* = 93, Stage III/IV *n* = 19, expansile *n* = 75, infiltrative n = 11, Grade 1 *n* = 48, Grade 2/3 *n* = 54.

**Table 3:** Association between immune cell phenotypes and overall survival.

		TUMOR						STROMA	
		Univariate			Multivariable*			Univariate	
	n	HR (95% CI)	p-value	n	HR (95% CI)	p-value	n	HR (95% CI)	p-value
**CD8+ FOXP3+**	**105**	**7.80 (2.48–24.47)**	**4.36 × 10** ^**−4**^	104	0.84 (0.17–4.15)	0.832	97	n/a	0.997
**CD8− FOXP3+**	**105**	**2.35 (1.15–4.78)**	**0.019**	104	1.60 (0.58–4.36)	0.362	97	1.04 (0.61–1.77)	0.882
**CD68+ PDL1−**	**114**	**1.89 (1.08–3.32)**	**0.026**	111	1.43 (0.78–2.61)	0.248	109	1.82 (0.95–3.49)	0.071
**Plasma cell**	**102**	**0.53 (0.30–0.94)**	**0.03**	100	0.53 (0.27–1.06)	0.07	102	1.47 (0.86–2.50)	0.156
CD4+ T cells	97	1.76 (0.98–3.17)	0.057				97	1.2 (0.76–1.89)	0.431
CD68+ PDL1+	114	1.84 (0.95–3.58)	0.070				109	1.12 (0.61–2.03)	0.72
PD1+	114	1.27 (0.72–2.22)	0.406				109	0.99 (0.67–1.48)	0.971
PDL1+	114	1.13 (0.69–1.87)	0.616				109	1.12 (0.72–1.75)	0.626
CD20+ B cells	102	0.91 (0.53–1.56)	0.736				102	0.82 (0.51–1.31)	0.402
CD8+ T cells	105	1.01 (0.64–1.59)	0.957				97	1.25 (0.87–1.80)	0.23

* Adjusted for age, stage, stratified by study site.

## HIGHLIGHTS

- High stage compared to low stage MOC have features suggestive of a more immunosuppressive environment.
- High epithelial FOXP3+ T-regulatory cells associated with poorer overall survival, possibly inhibiting the immune response.
- Most mucinous ovarian carcinomas are immunogenically ‘cold’ and tailored immune therapy approaches would be required.
