# Supplementary material for: Profiling the immune landscape in mucinous ovarian carcinoma
Source: Gynecol Oncol. Author manuscript; Available in PMC 2023 Jul 27. (PMC10374276; doi:10.1016/j.ygyno.2022.10.022)

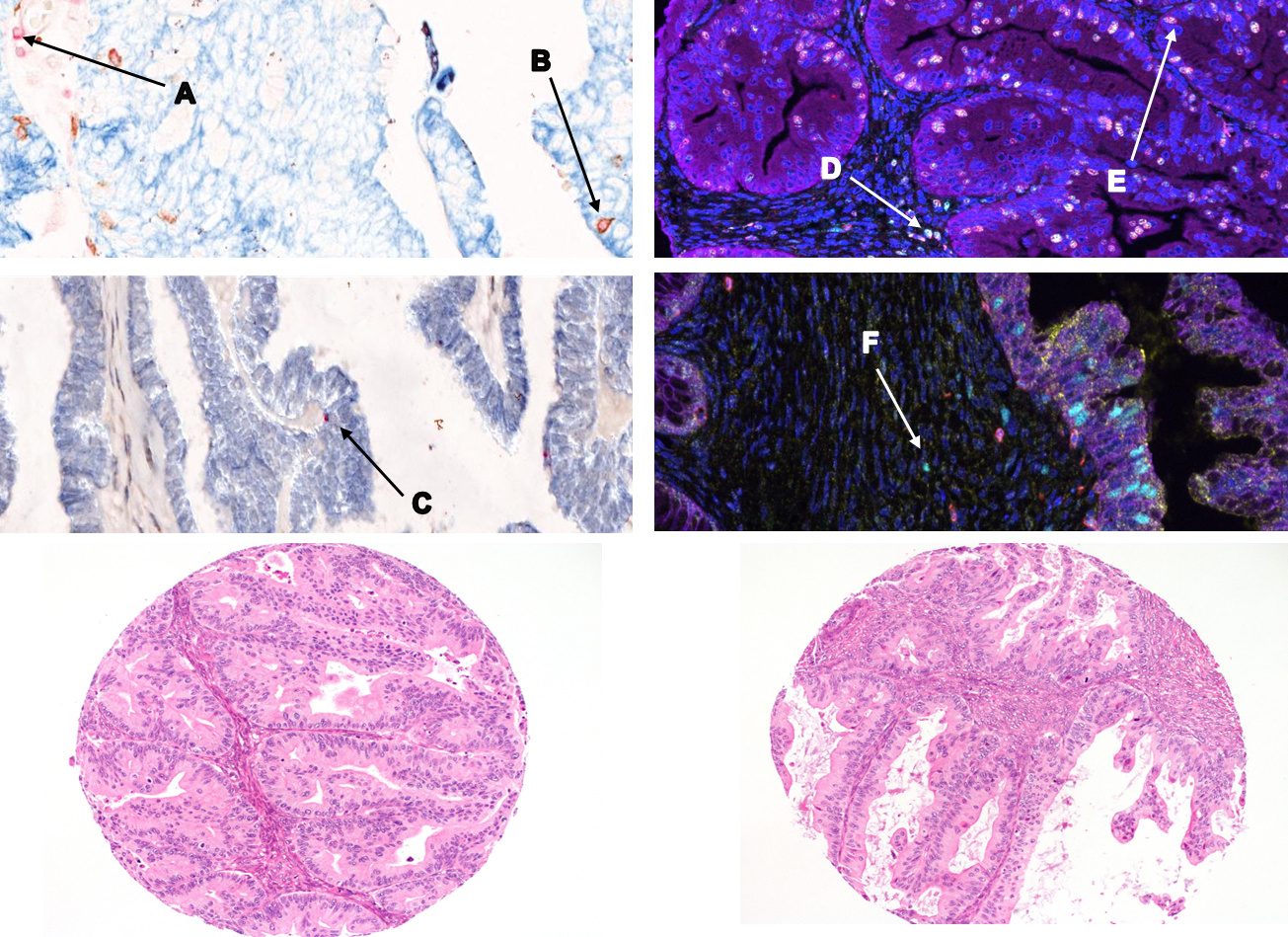
Supplementary Figure S1: Sample images of cores of brightfield IHC and immunofluorescence. A) CD4+ T cell in the stroma; B) CD8+ T cell in tumor epithelium; C) CD79a+ plasma cell in the tumor epithelium D) PD-L1+ macrophage in stroma; E) PD-L1- macrophage in tumor epithelium; F) FOXP3+ putative T-regulatory cell in the stroma; G,H) representative hematoxylin and eosin images of mucinous ovarian carcinoma.

**H**

**G**

**D**

Supplementary Figure S2: Boxplots of tumor epithelial and stromal densities by study site before batch correction, immune fluorescent tumor-associated macrophage/PD-L1/PD-1 panel. Some panels have a split scale due to outliers.


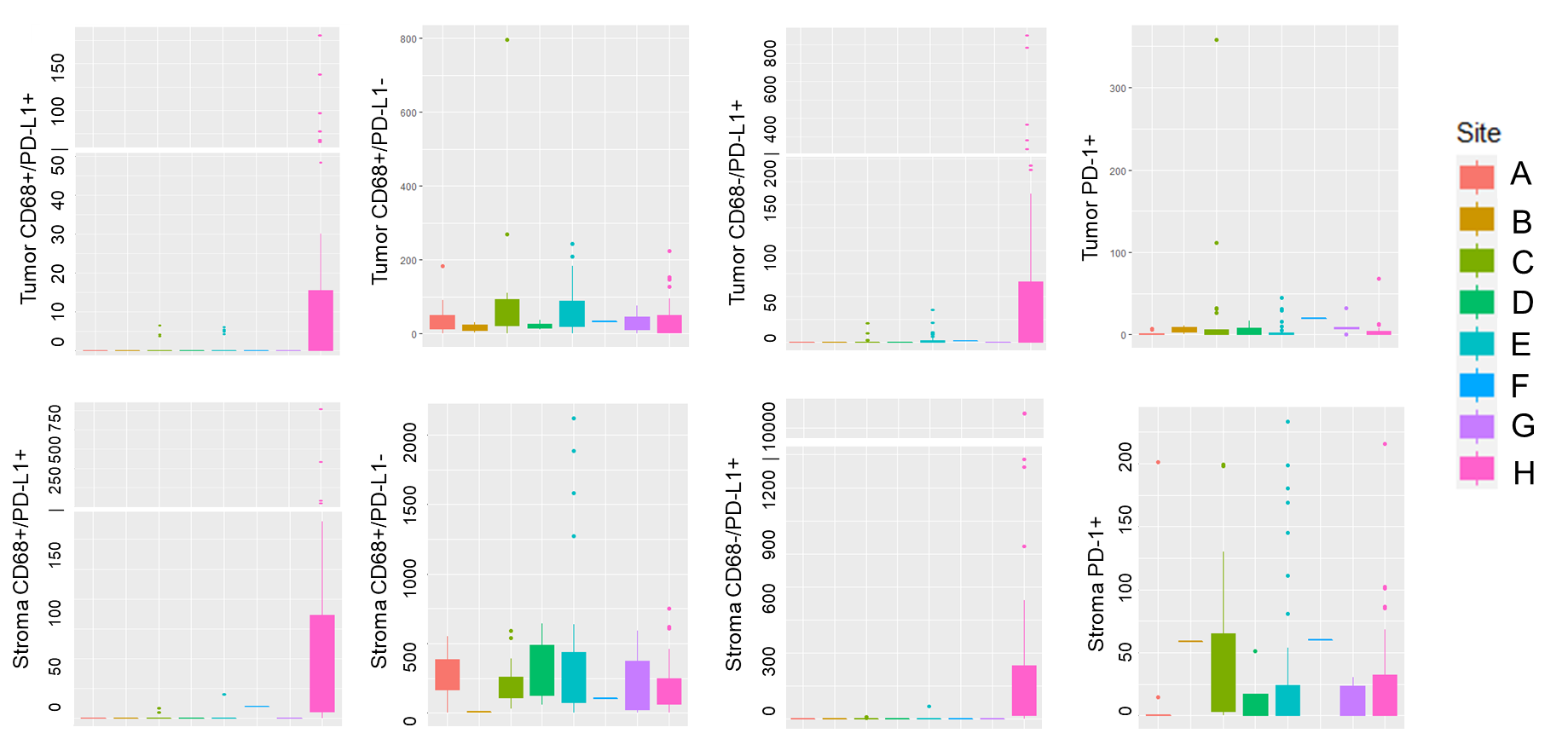


Supplementary Figure S3: Boxplots of tumor epithelial and stromal densities by study site before batch correction, immune fluorescent T-regulatory/ CD8 T-cell panel.


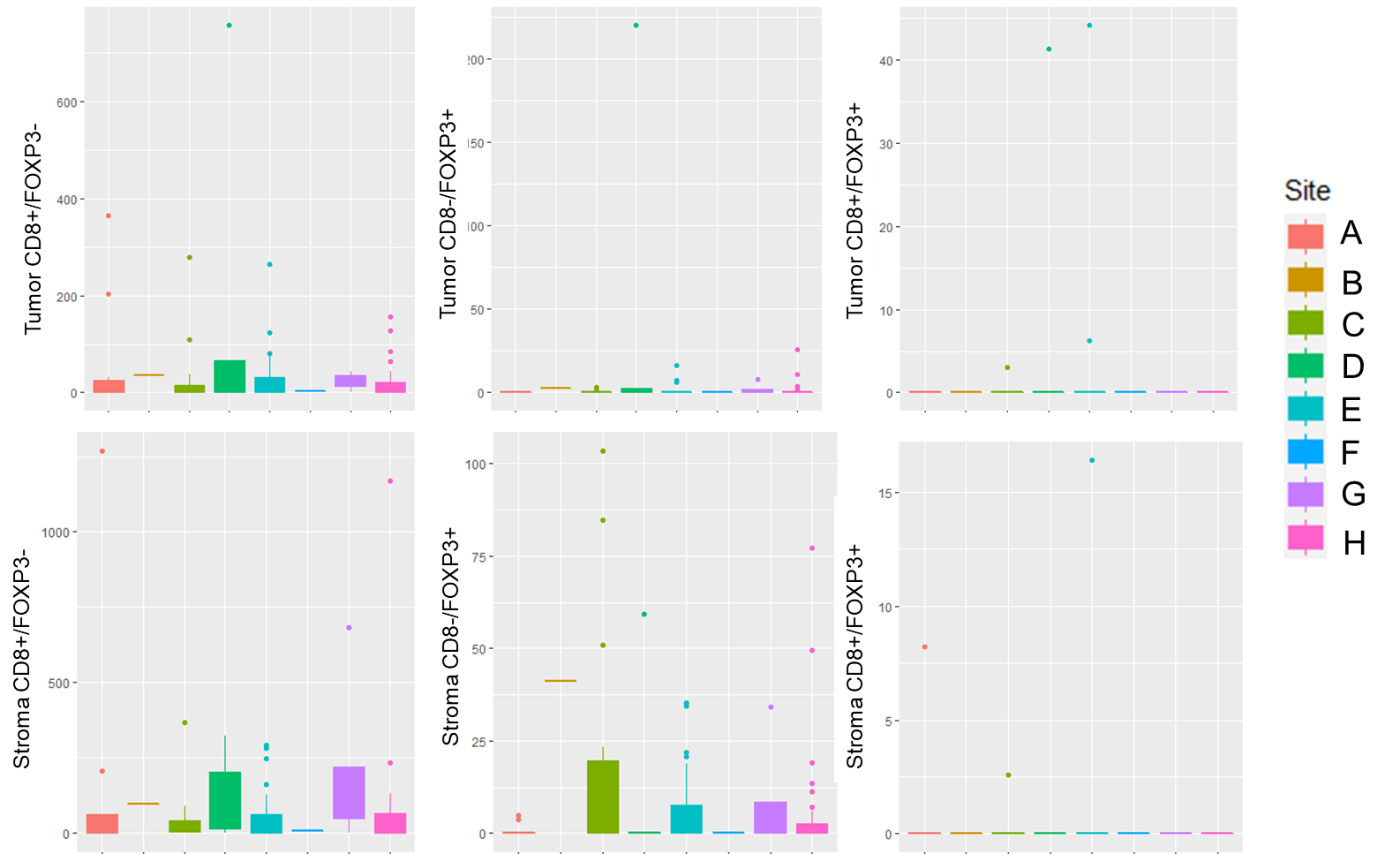


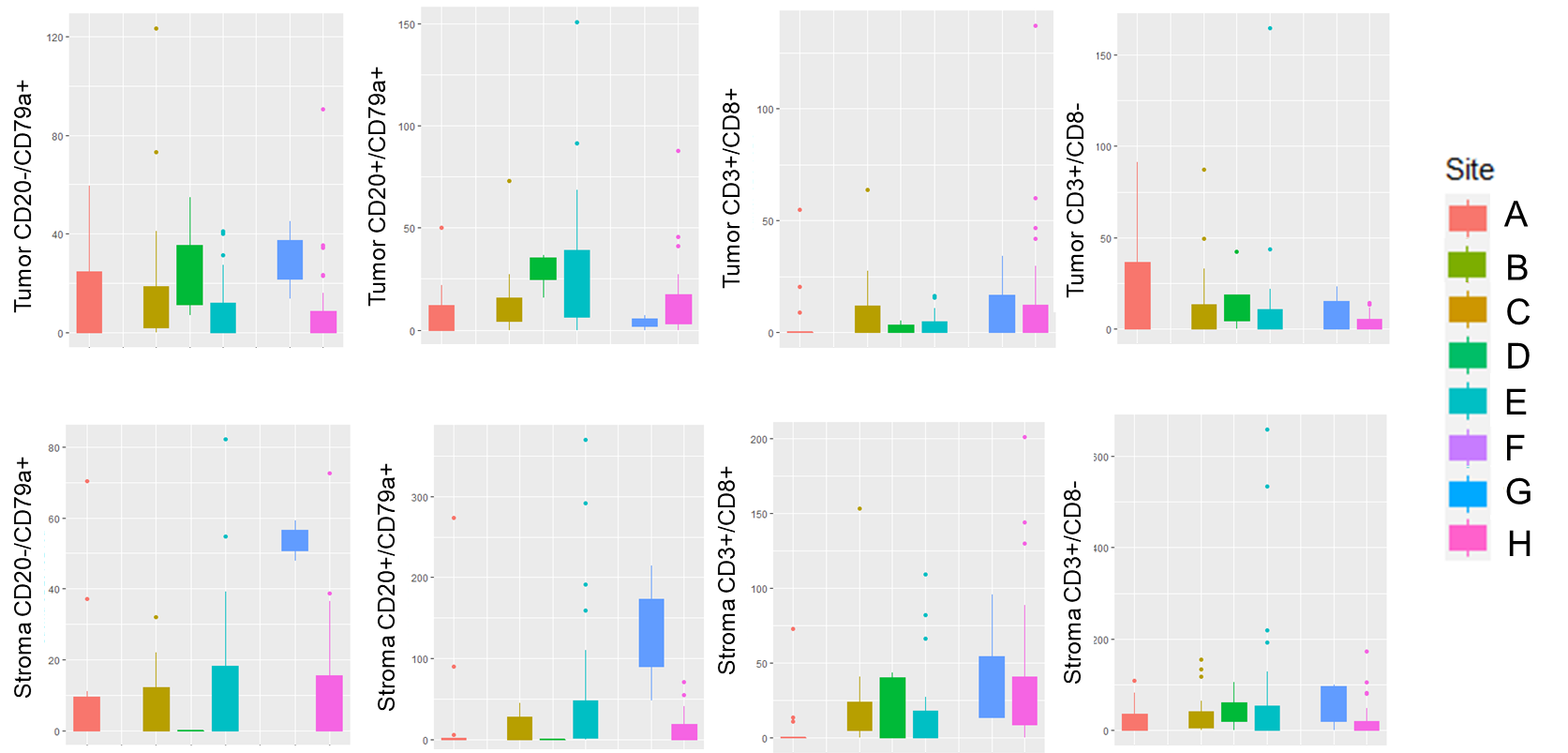
Supplementary Figure S4: Boxplots of tumor epithelial and stromal densities by study site before batch correction (sites B and G not scored), immunohistochemistry T cell and B cell panels.

Supplementary Figure S5: Boxplots of non-significant differences in density in immune phenotypes by tumor epithelium/stromal regions. Difference in mean density calculated using Welch’s t-test.


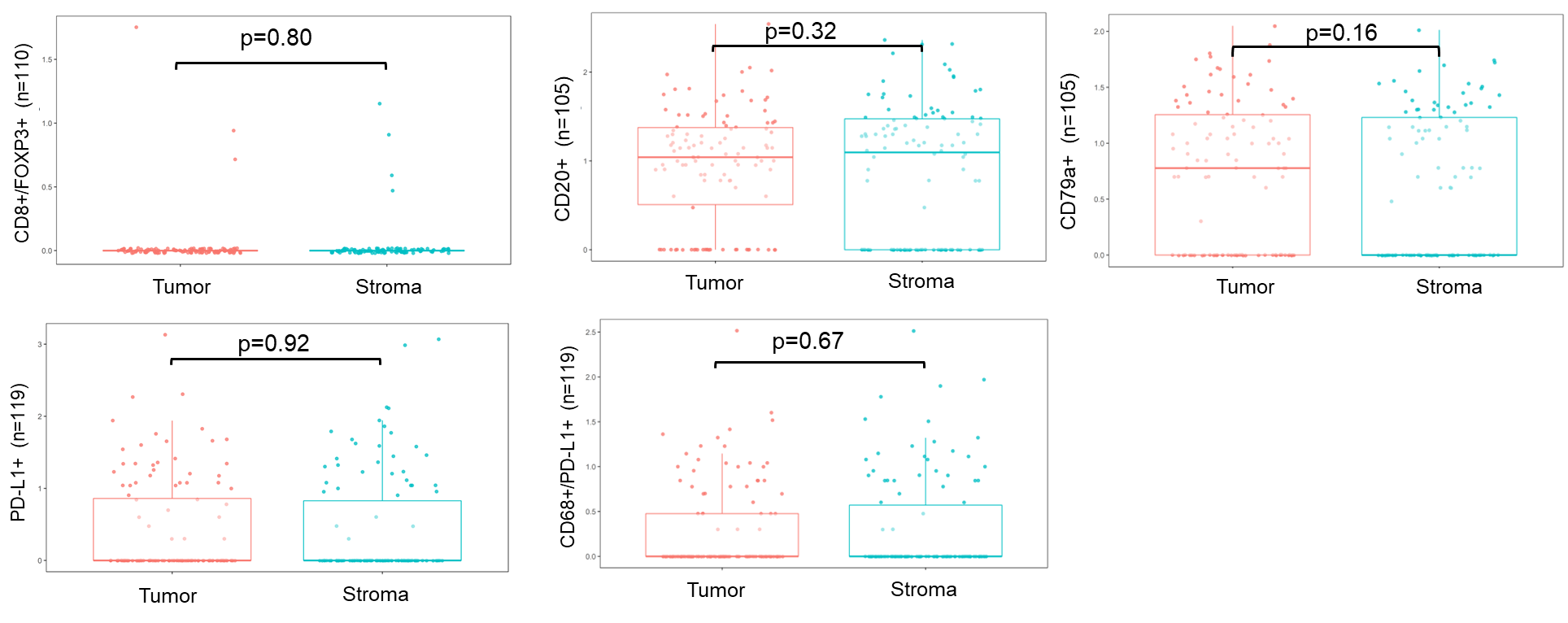


Supplementary Figure S6: Unsupervised kmeans clustering of cell densities in tumor epithelium and stroma, restricted to FIGO Stage IC-IV (n=33).


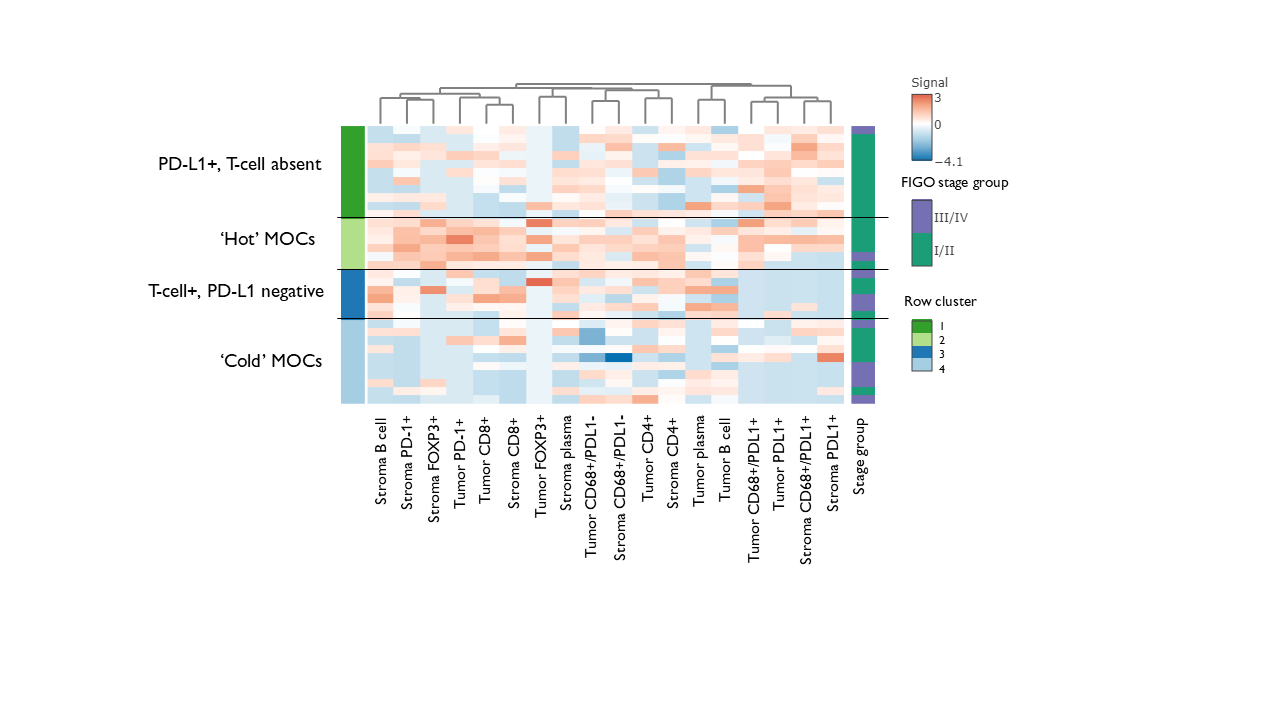

Supplement: 1 [file NIHMS1916158-supplement-1.docx]
