# Supplementary material for: Profiling the immune landscape in mucinous ovarian carcinoma
Source: Gynecol Oncol. Author manuscript; Available in PMC 2023 Jul 27. (PMC10374276; doi:10.1016/j.ygyno.2022.10.022)
Supplement: 2 [file NIHMS1916158-supplement-2.docx]

| Supplementary Table S1: Ovarian Tumor Tissue Analysis consortium studies | | | | |  |  |
| --- | --- | --- | --- | --- | --- | --- |
| **Study site** | **Name** | **Location** | **Years** | **Ascertainment of Patients and Clinical Data** | **Ethics committee** | **Informed consent** |
| BRZ | Brazil Gynecologic Tumor Bank (BRZ) study | Brazil | 1987-2010 | University Hospital of Ribeirao Preto School of Medicine (HCRP), case series with prospective follow up | Research Ethics Committee of Hospital das Clínicas of the Ribeirão Preto Medical School | No / pathology material |
| CNI | CNIO Ovarian Cancer Study | Spain | 2006-2013 | Hospitals in Madrid in Medical Oncology Divisions | Bioethics and Animal Welfare Committee of the Carlos III Health Institute | Yes |
| DOV | Diseases of the Ovary and their Evaluation | USA | 2002-2009 | 13 counties from western Washington SEER registry | Fred Hutchinson Cancer Research Center Institutional Review Board | Yes |
| GER | Germany Ovarian Cancer Study | Germany | 1993-1996 | 26 hospitals in the study regions | Ethics Committee of the Heidelberg University Clinic | Yes |
| SEA | Study of Epidemiology and Risk Factors in Cancer Heredity | UK | 1998-present | Eastern Region Cancer Intelligence Unit, West Midlands Cancer Intelligence Unit, and multiple cancer networks | Cambridgeshire 4 Research Ethics Committee | Yes |
| TVA | Ovarian Cancer in Alberta | Canada | 2005-2011 | Alberta Cancer Registry and affiliated hospitals | Health Research Ethics Board of Alberta | Yes |
| UKO | United Kingdom Ovarian Cancer Population study | UK | 2006-2010 | Ten major Gynecologic Oncology NHS centers in England, Wales and Northern Ireland; cancer registries; NHS Information Centre for Health and Social Care (England and Wales) and Central Services Agency (Northern Ireland) | National Health Service Central Office for Research Ethics Committees (COREC) and The Joint University College London/University College London Hospital Committee on the Ethics of Human Research (Committee A) | Yes |
| VAN | Vancouver Ovarian Cancer Study | Canada | 1984-2000 | Ovarian Cancer Registry serving British Columbia and the Cheryl Brown Outcomes Unit | University of British Columbia - British Columbia Cancer Agency Research Ethics Board | Some cases Yes and some cases No / pathology material |

| Supplementary Table S2: Cell phenotypes in each panel | | |  |
| --- | --- | --- | --- |
| Method | Phenotype | Cell type | Cases for analysis (n) |
| Immune fluorescence | CD68+ PDL1- | PD-L1 negative macrophages | 119 |
| Immune fluorescence | CD68+ PDL1+ | PD-L1 positive macrophages | 119 |
| Immune fluorescence | PD1+ | PD-1 positive cells | 119 |
| Immune fluorescence | CD68- PDL1+ | PD-L1 positive cells | 119 |
| Immune fluorescence | CD8+ FOXP3- | CD8 T cells | 110 |
| Immune fluorescence | FOXP3+ CD25- | FOXP3 T-regulatory cells | 110 |
| Immune fluorescence | FOXP3+ CD25+ | FOXP3 T-regulatory cells | 110 |
| Immune fluorescence | CD8+ FOXP3+ | CD8 positive FOXP3 positive cells | 110 |
| Brightfield immunohistochemistry | CD20- CD79a+ | Plasma cells | 109 |
| Brightfield immunohistochemistry | CD20+ CD79a+ | CD20+ B cells | 109 |
| Brightfield immunohistochemistry | CD3+ CD8- | CD4 T cells | 100 |
| Brightfield immunohistochemistry | CD3+ CD8+ | CD8 T cells | 100 |

| Supplementary Table S3: Primary antibodies used for immunohistochemistry and immune fluorescence | | | | |  |
| --- | --- | --- | --- | --- | --- |
| **Antigen** | **Supplier** | **Cat. #** | **Clone** | **Dilution** |  |
| Pan Cytokeratin | Biocare | CM162 | AE1/AE3 + 5D3 | 1/400 DVG |  |
| CD3 | Spring Bioscience | M3074 | SP7 | 1/500 DVG |  |
| CD8 | Cell Marque | 108M-94 | C8/144B | 1/250 DVG |  |
| CD20 | Biocare | CM004 | L26 | 1/400 DVG |  |
| CD79a | Abcam | Ab16698 | SP18 | 1/400 DVG |  |
| FOXP3 | Abcam | Ab20034 | 236A/E7 | 1/200 DVG |  |
| CD25 | Labvision | MS-1088 | 4C9 | 1/800 DVG |  |
| PD-L1 | Spring Bioscience | MY422 | SP142 | 1/200 DVG |  |
| PD-1 | Abcam | Ab137132 | EPr4877 2 | 1/300 DVG |  |
| CD68 | Spring Bioscience | M5512 | SP251 | 1/350 DVG |  |

| Supplementary Table S4: Valid counts for each core across stromal/tumor epithelial regions of each panel | | | | | | | | | |
| --- | --- | --- | --- | --- | --- | --- | --- | --- | --- |
| core_id | | IF TREG/CD8 stroma | IF TREG/CD8 tumor | IF TAM/PD-1/PD-L1 stroma | IF TAM/PD-1/PD-L1 tumor | IHC T cell stroma | IHC T cell tumor | IHC B cell stroma | IHC B cell tumor |
| 1 | Yes | | Yes | Yes | Yes | Yes | Yes | Yes | Yes |
| 2 | Yes | | Yes | Yes | Yes | Yes | Yes | Yes | Yes |
| 3 | Yes | | Yes | Yes | Yes | Yes | Yes | Yes | Yes |
| 4 | Yes | | Yes | Yes | Yes | Yes | Yes | Yes | Yes |
| 5 | Yes | | Yes | Yes | Yes | Yes | Yes | Yes | Yes |
| 6 | Yes | | Yes | Yes | Yes | Yes | Yes | Yes | Yes |
| 7 | Yes | | Yes | Yes | Yes | Yes | Yes | Yes | Yes |
| 8 | Yes | | Yes | Yes | Yes | Yes | Yes | Yes | Yes |
| 9 | Yes | | Yes | Yes | Yes | Yes | Yes | Yes | Yes |
| 10 | Yes | | Yes | Yes | Yes | Yes | Yes | Yes | Yes |
| 11 | Yes | | Yes | Yes | Yes | Yes | Yes | Yes | Yes |
| 12 | Yes | | Yes | Yes | Yes | Yes | Yes | Yes | Yes |
| 13 | Yes | | Yes | Yes | Yes | Yes | Yes | Yes | Yes |
| 14 | Yes | | Yes | Yes | Yes | Yes | Yes | Yes | Yes |
| 15 | Yes | | Yes | Yes | Yes | Yes | Yes | Yes | Yes |
| 16 | Yes | | Yes | Yes | Yes | Yes | Yes | Yes | Yes |
| 17 | Yes | | Yes | Yes | Yes | Yes | Yes | Yes | Yes |
| 18 | Yes | | Yes | Yes | Yes | Yes | Yes | Yes | Yes |
| 19 | Yes | | Yes | Yes | Yes | Yes | Yes | Yes | Yes |
| 20 | Yes | | Yes | Yes | Yes | Yes | Yes | Yes | Yes |
| 21 | Yes | | Yes | Yes | Yes | Yes | Yes | Yes | Yes |
| 22 | Yes | | Yes | Yes | Yes | Yes | Yes | Yes | Yes |
| 23 | Yes | | Yes | Yes | Yes | Yes | Yes | Yes | Yes |
| 24 | Yes | | Yes | Yes | Yes | Yes | Yes | Yes | Yes |
| 25 | Yes | | Yes | Yes | Yes | Yes | Yes | Yes | Yes |
| 26 | Yes | | Yes | Yes | Yes | Yes | Yes | Yes | Yes |
| 27 | Yes | | Yes | Yes | Yes | Yes | Yes | Yes | Yes |
| 28 | Yes | | Yes | Yes | Yes | Yes | Yes | Yes | Yes |
| 29 | Yes | | Yes | Yes | Yes | Yes | Yes | Yes | Yes |
| 30 | Yes | | Yes | Yes | Yes | Yes | Yes | Yes | Yes |
| 31 | Yes | | Yes | Yes | Yes | Yes | Yes | Yes | Yes |
| 32 | Yes | | Yes | Yes | Yes | Yes | Yes | Yes | Yes |
| 33 | Yes | | Yes | Yes | Yes | Yes | Yes | Yes | Yes |
| 34 | Yes | | Yes | Yes | Yes | Yes | Yes | Yes | Yes |
| 35 | Yes | | Yes | Yes | Yes | Yes | Yes | Yes | Yes |
| 36 | Yes | | Yes | Yes | Yes | Yes | Yes | Yes | Yes |
| 37 | Yes | | Yes | Yes | Yes | Yes | Yes | Yes | Yes |
| 38 | Yes | | Yes | Yes | Yes | Yes | Yes | Yes | Yes |
| 39 | Yes | | Yes | Yes | Yes | Yes | Yes | Yes | Yes |
| 40 | Yes | | Yes | Yes | Yes | Yes | Yes | Yes | Yes |
| 41 | Yes | | Yes | Yes | Yes | Yes | Yes | Yes | Yes |
| 42 | Yes | | Yes | Yes | Yes | Yes | Yes | Yes | Yes |
| 43 | Yes | | Yes | Yes | Yes | Yes | Yes | Yes | Yes |
| 44 | Yes | | Yes | Yes | Yes | Yes | Yes | Yes | Yes |
| 45 | Yes | | Yes | Yes | Yes | Yes | Yes | Yes | Yes |
| 46 | Yes | | Yes | Yes | Yes | Yes | Yes | Yes | Yes |
| 47 | Yes | | Yes | Yes | Yes | Yes | Yes | Yes | Yes |
| 48 | Yes | | Yes | Yes | Yes | Yes | Yes | Yes | Yes |
| 49 | Yes | | Yes | Yes | Yes | Yes | Yes | Yes | Yes |
| 50 | Yes | | Yes | Yes | Yes | Yes | Yes | Yes | Yes |
| 51 | Yes | | Yes | Yes | Yes | Yes | Yes | Yes | Yes |
| 52 | Yes | | Yes | Yes | Yes | Yes | Yes | Yes | Yes |
| 53 | Yes | | Yes | Yes | Yes | Yes | Yes | Yes | Yes |
| 54 | Yes | | Yes | Yes | Yes | Yes | Yes | Yes | Yes |
| 55 | Yes | | Yes | Yes | Yes | Yes | Yes | Yes | Yes |
| 56 | Yes | | Yes | Yes | Yes | Yes | Yes | Yes | Yes |
| 57 | Yes | | Yes | Yes | Yes | Yes | Yes | Yes | Yes |
| 58 | Yes | | Yes | Yes | Yes | Yes | Yes | Yes | Yes |
| 59 | Yes | | Yes | Yes | Yes | Yes | Yes | Yes | Yes |
| 60 | Yes | | Yes | Yes | Yes | Yes | Yes | Yes | Yes |
| 61 | Yes | | Yes | Yes | Yes | Yes | Yes | Yes | Yes |
| 62 | Yes | | Yes | Yes | Yes | Yes | Yes | Yes | Yes |
| 63 | Yes | | Yes | Yes | Yes | Yes | Yes | Yes | Yes |
| 64 | Yes | | Yes | Yes | Yes | Yes | Yes | Yes | Yes |
| 65 | Yes | | Yes | Yes | Yes | Yes | Yes | Yes | Yes |
| 66 | Yes | | Yes | Yes | Yes | Yes | Yes | Yes | Yes |
| 67 | Yes | | Yes | Yes | Yes | Yes | Yes | Yes | Yes |
| 68 | Yes | | Yes | Yes | Yes | Yes | Yes | Yes | Yes |
| 69 | Yes | | Yes | Yes | Yes | Yes | Yes | Yes | Yes |
| 70 | Yes | | Yes | Yes | Yes | Yes | Yes | Yes | Yes |
| 71 | Yes | | Yes | Yes | Yes | Yes | Yes | Yes | Yes |
| 72 | Yes | | Yes | Yes | Yes | Yes | Yes | Yes | Yes |
| 73 | Yes | | Yes | Yes | Yes | Yes | Yes | Yes | Yes |
| 74 | Yes | | Yes | Yes | Yes | Yes | Yes | Yes | Yes |
| 75 | Yes | | Yes | Yes | Yes | Yes | Yes | Yes | Yes |
| 76 | Yes | | Yes | Yes | Yes | Yes | Yes | Yes | Yes |
| 77 | Yes | | Yes | Yes | Yes | Yes | Yes |  |  |
| 78 | Yes | | Yes | Yes | Yes | Yes | Yes |  |  |
| 79 | Yes | | Yes | Yes | Yes | Yes | Yes |  |  |
| 80 | Yes | | Yes | Yes | Yes | Yes | Yes |  |  |
| 81 | Yes | | Yes | Yes | Yes | Yes | Yes |  |  |
| 82 | Yes | | Yes | Yes | Yes | Yes | Yes |  |  |
| 83 | Yes | | Yes | Yes | Yes | Yes | Yes |  |  |
| 84 | Yes | | Yes | Yes | Yes |  |  | Yes | Yes |
| 85 | Yes | | Yes | Yes | Yes |  |  | Yes | Yes |
| 86 | Yes | | Yes | Yes | Yes |  |  | Yes | Yes |
| 87 | Yes | | Yes | Yes | Yes |  |  | Yes | Yes |
| 88 | Yes | | Yes | Yes | Yes |  |  | Yes | Yes |
| 89 | Yes | | Yes | Yes | Yes |  |  | Yes | Yes |
| 90 | Yes | | Yes | Yes | Yes |  |  | Yes | Yes |
| 91 | Yes | | Yes | Yes | Yes |  |  | Yes | Yes |
| 92 | Yes | | Yes | Yes | Yes |  |  | Yes | Yes |
| 93 | Yes | | Yes | Yes | Yes |  |  |  |  |
| 94 | Yes | | Yes | Yes | Yes |  |  |  |  |
| 95 | Yes | | Yes | Yes | Yes |  |  |  |  |
| 96 | Yes | | Yes |  | Yes | Yes | Yes | Yes | Yes |
| 97 | Yes | | Yes |  | Yes | Yes | Yes | Yes | Yes |
| 98 | Yes | | Yes |  | Yes | Yes | Yes | Yes | Yes |
| 99 | Yes | | Yes |  | Yes | Yes | Yes |  |  |
| 100 | Yes | | Yes |  |  | Yes | Yes | Yes | Yes |
| 101 | Yes | | Yes |  |  |  |  |  |  |
| 102 | Yes | | Yes |  |  |  |  |  |  |
| 103 |  | | Yes | Yes | Yes | Yes | Yes | Yes | Yes |
| 104 |  | | Yes | Yes | Yes | Yes | Yes | Yes | Yes |
| 105 |  | | Yes | Yes | Yes | Yes | Yes | Yes | Yes |
| 106 |  | | Yes | Yes | Yes | Yes | Yes | Yes | Yes |
| 107 |  | | Yes | Yes | Yes |  |  | Yes | Yes |
| 108 |  | | Yes | Yes | Yes |  |  |  |  |
| 109 |  | | Yes | Yes | Yes |  |  |  |  |
| 110 |  | | Yes | Yes | Yes |  |  |  |  |
| 111 |  | |  | Yes | Yes | Yes | Yes | Yes | Yes |
| 112 |  | |  | Yes | Yes | Yes | Yes | Yes | Yes |
| 113 |  | |  | Yes | Yes | Yes | Yes | Yes | Yes |
| 114 |  | |  | Yes | Yes | Yes | Yes | Yes | Yes |
| 115 |  | |  | Yes | Yes | Yes | Yes |  |  |
| 116 |  | |  | Yes | Yes | Yes | Yes |  |  |
| 117 |  | |  | Yes | Yes |  |  | Yes | Yes |
| 118 |  | |  | Yes | Yes |  |  | Yes | Yes |
| 119 |  | |  | Yes | Yes |  |  |  |  |
| 120 |  | |  | Yes | Yes |  |  |  |  |
| 121 |  | |  | Yes | Yes |  |  |  |  |
| 122 |  | |  |  | Yes |  |  | Yes | Yes |
| 123 |  | |  |  |  | Yes | Yes | Yes | Yes |
| 124 |  | |  |  |  | Yes | Yes | Yes | Yes |
| 125 |  | |  |  |  | Yes | Yes | Yes | Yes |
| 126 |  | |  |  |  | Yes | Yes | Yes | Yes |

| Supplementary Table S5: Patient and tumor characteristics by panel | | | | |
| --- | --- | --- | --- | --- |
|  | CD20/CD79a | CD3/CD8 | CD68/PD-L1/PD-1 | FOXP3/CD8 |
| Total n | 105 | 100 | 119 | 110 |
| Age (median) | 53 | 53 | 54 | 53 |
| Stage I/II | 84 (80%) | 80 (80%) | 96 (81%) | 90 (82%) |
| Stage III/IV | 19 (18%) | 18 (18%) | 19 (16%) | 18 (16%) |
| Stage unknown | 2 (2%) | 2 (2%) | 4 (3%) | 2 (2%) |
| Expansile | 71 (68%) | 66 (66%) | 75 (63%) | 70 (64%) |
| Infiltrative | 11 (10%) | 10 (10%) | 15 (13%) | 12 (11%) |
| Unknown pattern | 23 (22%) | 24 (24%) | 29 (24%) | 28 (25%) |
| Grade 1 | 43 (41%) | 44 (44%) | 51 (43%) | 48 (44%) |
| Grade 2 | 46 (44%) | 41 (41%) | 51 (43%) | 45 (41%) |
| Grade 3 | 4 (4%) | 5 (5%) | 5 (4%) | 5 (5%) |
| Grade unknown | 12 (11%) | 10 (10%) | 12 (10%) | 12 (11%) |

| Supplementary Table S6: Associations between immune cell phenotypes and pattern of invasion for FIGO Stage I | | |
| --- | --- | --- |
|  |  |  |
| CD68+ PDL1- |  |  |
| epithelium | 0.43 |  |
| stroma | 0.51 |  |
| CD68+ PDL1+ |  |  |
| epithelium | 0.44 |  |
| stroma | 0.24 |  |
| PDL1+ |  |  |
| epithelium | 0.67 |  |
| stroma | 0.07 |  |
| PD1+ |  |  |
| epithelium | 0.46 |  |
| stroma | 0.07 |  |
| CD3+/CD8+ (CD4+) T cells |  |  |
| epithelium | 0.87 |  |
| stroma | 0.89 |  |
| CD8+ T cells |  |  |
| epithelium | 0.67 |  |
| stroma | 0.44 |  |
| CD8+ FOXP3+ |  |  |
| epithelium | 0.32 |  |
| stroma | 0.57 |  |
| CD8- FOXP3+ |  |  |
| epithelium | 0.24 |  |
| stroma | 0.57 |  |
| CD20+ B cell |  |  |
| epithelium | 0.73 |  |
| stroma | 0.97 |  |
| CD79a+ plasma cell |  |  |
| epithelium | 0.06 |  |
| stroma | 0.53 |  |
|  |  |  |
| p-values calculated using Welch t-test | |  |

| Supplementary Table S7: Tumor stage by cluster and association with overall survival | | | | | | |  |  |  |
| --- | --- | --- | --- | --- | --- | --- | --- | --- | --- |
|  |  | TOTAL | FIGO stage III/IV patients | | Survival data | Univariate | | Multivariable | |
|  | Phenotype | n | n | % | n | HR (95% CI) | p-value | HR (95% CI) | p-value |
| Cluster 1 | PD-L1-/T cell+ | 14 | 3 | 21% | 13 | ref | | ref | |
| Cluster 2 | PD-L1+/T cell+ | 11 | 1 | 9% | 11 | 1.88 (0.42-9.40) | 0.41 | 1.91 (0.32-11.54) | 0.479 |
| Cluster 3 | PD-L1+/T cell- | 18 | 2 | 11% | 18 | 2.40 (0.63-9.01) | 0.198 | 2.41 (0.34-17.25) | 0.381 |
| Cluster 4 | PD-L1-/T cell- | 33 | 6 | 18% | 28 | 0.86 (0.20-3.59) | 0.834 | 0.79 (0.17-3.56) | 0.755 |

Supplementary Table S8: Sensitivity analysis of associations between tumor epithelial densities and overall survival, removing high PD-L1 density study

|  |  | univariate | |
| --- | --- | --- | --- |
|  | n | HR (95% CI) | p-value |
| CD8+ FOXP3+ | 68 | 1.05 (1.01-1.10) | 9.57E-03 |
| CD8- FOXP3+ | 68 | 1.01 (1.00-1.02) | 0.008 |
| CD68+ PDL1- | 77 | 1.01 (1.00-1.10) | 0.002 |
| Plasma cell | 68 | 0.69 (0.35-1.36 | 0.286 |
| CD4+ T cells | 64 | 2.52 (1.81-5.38) | 0.017 |
| CD68+ PDL1+ | 77 | 1.07 (0.96-1.20) | 0.214 |
| PD1+ | 77 | 1.01 (1.00-1.02 | 0.029 |
| PDL1+ | 77 | 0.99 (0.93-1.05) | 0.702 |
| CD20+ B cells | 68 | 1.01 (0.52-1.95) | 0.981 |
| CD8+ T cells | 68 | 1.00 (1.00-1.01) | 0.036 |
